# Parameter and state estimation of backers yeast cultivation with a gas sensor array and unscented Kalman filter

**DOI:** 10.1002/elsc.202000058

**Published:** 2020-12-04

**Authors:** Abdolrahimahim Yousefi‐Darani, Olivier Paquet‐Durand, Jörg Hinrichs, Bernd Hitzmann

**Affiliations:** ^1^ Department of Process Analytics and Cereal Science University of Hohenheim Stuttgart Germany; ^2^ Department of Soft Matter Science and Dairy Technology University of Hohenheim Stuttgart Germany

**Keywords:** batch cultivation, bioprocess supervision, ethanol, state estimation, Unscented Kalman filter

## Abstract

Real‐time information about the concentrations of substrates and biomass is the key to accurate monitoring and control of bioprocess. However, on‐line measurement of these variables is a challenging task and new measurement systems are still required. An alternative are software sensors, which can be used for state and parameter estimation in bioprocesses. The software sensors predict the state of the process by using mathematical models as well as data from measured variables. The Kalman filter is a type of such sensors.

In this paper, we have used the Unscented Kalman Filter (UKF) which is a nonlinear extension of the Kalman filter for on‐line estimation of biomass, glucose and ethanol concentration as well as for estimating the growth rate parameters in *S. cerevisiae* batch cultivation, based on infrequent ethanol measurements. The UKF algorithm was validated on three different cultivations with variability of the substrate concentrations and the estimated values were compared to the off‐line values.

The results obtained showed that the UKF algorithm provides satisfactory results with respect to estimation of concentrations of substrates and biomass as well as the growth rate parameters during the batch cultivation.

**Abbreviations**
EKF: Extended Kalman filter
*S. cerevisiae*: *Saccharomyces cerevisiae*
UKF: unscented Kalman filter

## 1 INTRODUCTION

The ability to measure primary process variables, such as biomass, substrate and product concentrations is essential in order to guarantee the successful operation and automatic control of bioprocesses at their optimal state. But direct on‐line measurements of these biological state variables are often not possible due to the lack of cheap or reliable measuring devices. In fact, in many practical applications, only some of the state variables involved are available for on‐line measurement. Therefore, the development of methodologies, namely software sensors which can provide accurate estimation of process variables that are not measurable in real time, is of great interest [2, 3, 4].

The classical Kalman filter and its nonlinear extensions are a type of such software sensors, which have received a huge interest for state estimation of bioprocesses. In general, the Kalman filters combine the information of a process model and the available measurements for state and parameter estimation. As Harvey [5] pointed out, in the classical Kalman filter, both the process model and the measurement equation of the state space model are linear. However, most bioprocesses are highly nonlinear, therefore the classical Kalman filter cannot be used for state estimation in such processes. Different nonlinear extensions of the Kalman filter are available which mostly differ in the approximation of the prediction uncertainty. The extended Kalman filter (EKF) is a standard nonlinear estimation technique which can handle nonlinearity's by local first order Taylor approximations of the non‐linear functions in the model. Implementation of EKF algorithms for state and parameter estimation in bioprocess have been reported in numerous studies. Lisci and Tronci et al. [6] have implemented an extended Kalman filter for state estimation in fed‐batch cultivation of *S. cerevisiae based on* temperature, dissolved oxygen and substrate concentration measurements. Krishna et al. [7] have implimented an EKF algorithm for estimation of lactose concentration in fed‐batch cultivation of *Kluyveromyces marxianus* based on dissolved oxygen measurements; Krämer and King [8] used an EKF for estimation of substrate and biomass concentration in fed‐batch cultivation of *S. cerevisiae*; Lee et al. [9] applied an EKF algorithm for noise filtering from the dissolved oxygen measurements during batch cultivation of *E. coli* and Hitzmann et al. [10] implemented an EKF complemented by a special flow‐injection analysis system for glucose measurements during fed‐batch cultivation of *S. cerevisiae*. Based on the estimation, a feed forward PI‐control with a set point of 0.5 g/L was carried out. The mean deviation of the set point and the estimated value as well as the set point and the measured value were 0.05 and 0.11 g/L respectively. A similar approach is discussed by Klockow et al. [11] where the time delay of the measurements was compensated by a ring‐buffer. They showed that a set point of 0.007 g/L can be realized reliably.

**PRACTICAL APPLICATION**

In a previous study we have designed and implemented a model‐based calibrated gas sensor array for on‐line measurement of ethanol concentration in batch cultivation with the yeast S. cerevisiae [1]. The obtained results indicate that the gas sensor array was able to predict ethanol concentration with high accuracy. However the predicted values are only available every five minutes. Therefore in this work, in order to have continues values of ethanol concentration as well as the values of biomass, glucose and the growth rates, we have implemented an unscented Kalman filter (UKF) algorithm.

The obtained results indicate, that accurate continues concentrations of the state variables as well as the growth rates can be obtained by the UKF algorithm. No off‐line measurements for calibration are required in the proposed algorithm.

The proposed method is a cheap alternative to other tools that are used for monitoring yeast cultivations such as spectroscopy based methods.

Usually the well‐known EKF shows good prediction results. Nevertheless, in spite of the reported satisfactory results, it has some disadvantages. It is reliable for systems which are almost linear on the time scale of the update intervals; it requires the calculation of Jacobians at each time step, which may be difficult to obtain for higher order systems; it does linear approximations of the system at a given time instant, which may introduce errors in the estimation, leading to a state divergence over time [12, 13].

The unscented Kalman filter (UKF) is another nonlinear extension of the classical Kalman filter which is very similar to the EKF, but instead of approximating the non‐linear process model by calculating the Jacobian of the dynamics for the determination of the estimation error variance, the transformed probability distributions are approximated directly. This is done by representing the distribution by a set of chosen sample points, transforming these points by the non‐linear model function, and then approximating the mean and variance of the transformed distribution by the mean and variance of the transformed points [14]. In recent years, a number of authors have demonstrate the successful application of UKF algorithms for state and parameter estimation in bioprocesses. For instance, Jianlin et al. [15] have implimented an UKF algorithm for biomass and substrate prediction based on dissolved oxygen and carbon dioxide measurements in a fed‐batch cultivation of *S. cerevisiae*. Using the same microorganism, Simutis and Lübbert [16] have applied an UKF algorithm for estimation of biomass and its specific growth rate based on oxygen uptake and CO_2_ formation rate measurements in a fed‐batch cultivation. Furthermore, Krämer and King [17] have used an UKF algorithm for filtering out noise from measured state variables which were predicted with a near infra‐red spectrometer in a fed‐batch cultivation of *S. cerevisiae*.

As it can be seen, previous studies have exclusively focused on implementing UKF algorithms in fed‐batch cultivations, however on‐line monitoring and estimation of state variables in batch cultivations is also crucial in order to achieve high productivity over the process. Therefore, in this contribution an UKF algorithm is designed for on‐line estimation of biomass, glucose and ethanol concentration as well as for estimating the growth rate parameters in *S. cerevisiae* batch cultivation, based on the infrequently available ethanol measurements. In order to evaluate the reliability of the UKF, the proposed algorithm was validated on three different cultivations with variability of the substrate concentrations.

This paper is organized as follows: In the coming section of this work, the experimental setup and the cultivation conditions, the dynamic model of *S. cerevisiae* batch cultivation and a brief description of the on‐line ethanol measurement method as well as the unscented Kalman filter are described. In section 3 results and discussion is presented, and section 4 concludes this paper.

## 2 MATERIALS AND METHODS

### 2.1 Batch cultivation process

Three batch cultivations of *S. cerevisiae*, named BC1, BC2, and BC3, were performed. *S. cerevisiae* (fresh baker's yeast, Oma's Ur‐Hefe) was pre‐cultivated before fermentation. 5 g of baker's yeast was used in all cultivations. The baker's yeast was inoculated into 100 mL Schatzmann medium [18] and after shaking for 10 min, they were added into the stainless steel tank bioreactor (Minifors, Inifors HT, Bottmingen, Switzerland). The medium used for batch cultivations was the same as for the pre‐culture, but with 9 g/L, 5 g/L and 2.85 g/L glucose for BC1, BC2, and BC3 respectively and 1 mL/L trace elements solution. All three batch runs were operated at the same conditions, that is, a constant temperature at 30°C and a maintained pH at 5. The aeration and agitation rates were kept constant at 3.5 L/min and 450 rpm, respectively. Detailed experimental conditions of the cultivations are described by Yousefi‐Darani et al. [1].

### 2.2 Nonlinear process model

For modelling the process, an ideal stirred tank reactor in batch mode has been assumed with a cell growth kinetic approximated by the Monod model, where the substrate glucose as well as ethanol (when glucose is depleted) are the single growth‐limiting factor. According to the mass balance, the dynamic process model consists of the following equations [19]:

(1) $$\frac{dX}{dt}=\;\mu_{G}X+\;\mu_{E}X$$

(2) $$\frac{dG}{dt}=-\frac{\mu_{G}X}{Y_{X/G}}$$

(3) $$\frac{dE}{dt}=\;\frac{\mu_{G}X}{Y_{E/G}}-\;\frac{\mu_{E}X}{Y_{X/E}}$$

were G, E and X are the glucose, ethanol and the biomass concentrations, respectively.$\;Y_{X/G}$, $Y_{E/G}$ and $Y_{X/E}$ are the yield coefficients with respect to the conversion from glucose to biomass, glucose to ethanol and ethanol to biomass, respectively. $\mu_{G}$ and $\mu_{E}$ are the specific growth rates on glucose and ethanol, respectively and are calculated as

(4) $$\mu_{G}=\;\frac{\mu_{max,\;G}\;\cdot \;G}{K_{G}+G}$$

(5) $$\mu_{E}=\;\frac{\mu_{max,\;E}\;\cdot \;E}{K_{E}+E}\;\cdot {\left(1-\;\frac{\mu_{G}}{\mu_{max,\;G}}\right)}^{2}$$

$\mu_{max,\;G}$ and $\mu_{max,\;E}$ are the maximum specific growth rates on glucose and on ethanol, respectively. The values for $\mu_{max,\;G}$ and $\mu_{max,\;E}$ are estimated with the UKF filtering.

Therefore they are described with two additional differential terms, which do not alter the values:

(6) $$\frac{d\mu_{max,\;G}\;}{dt}=\;0$$

(7) $$\frac{d\mu_{max,\;E}\;}{dt}=\;0$$

In this contribution, the yield coefficients as well as the Monod equation's constants (*K*_E_ and *K*_G_) have been fixed to values which were chosen from literature [1, 20].

### 2.3 On‐line ethanol measurements

**FIGURE 1:**
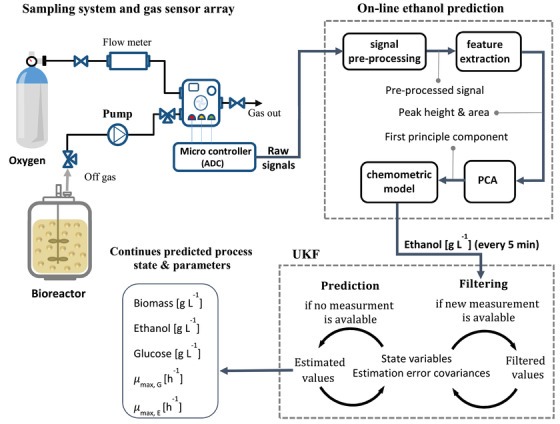
The operation scheme of the on‐line ethanol measurement system and the UKF algorithm for continuous state variables and parameter estimation

The on‐line ethanol measurements were performed in a self‐developed measurement system which contains two main parts, namely the headspace sampling system and the measurement chamber. Headspace sampling procedure consisted of an automated sequence of internal operations. First the head space samples of the bioreactor are pumped past the measurement chamber for 10 s at a flow rate of 400 mL min^−1^ with a diaphragm pump (Schwarzer Precision, Essen, Germany) every five minutes. The measurement chamber has a volume of 250 mL and contains a gas sensor array which is equipped with commercially available metal oxide semiconductor (MOS) gas sensors (TGS 822, TGS 813 and MQ3). In the next step the chamber is flushed by pure oxygen for regeneration. By preforming these steps, a peak shaped measurment signal is obtained. Ethanol concnetration is obtained from the raw signals by implementing signal processing methods and a chemometric model, which is described in detail in the literature [1]. Using the ethanol measurement in the gas phase, the ethanol concentration in the liquid phase of the cultivation broth is determined. Every 5 min a new ethanol measurement value is sent to the UKF algorithm. As measurement model the identity is used, i. e. the ethanol value itself. The operation scheme of the on‐line ethanol measurement system and the UKF algorithm is presented in Figure 1.

### 2.4 Unscented Kalman filter

In this work, an UKF is implemented to estimate continuous ethanol, glucose and biomass concentrations as well as the maximal growth rates during *S. cerevisiae* batch cultivation. As all Kalman filter approaches, the UKF calculates the most probable system state by appropriately weighing model predictions and actual measurements according to model uncertainty and measurement error, respectively. The UKF was chosen here over other extensions of the Kalman filter since it accurately calculates the statistical distributions of even nonlinear systems.

The UKF algorithms consist of two steps namely the prediction step (time update) and the filtering step (measurement update) which are summarized as follows:

- **Prediction step (time update)**:

Using the last known state ${\hat{X}}_{k}$ the process model is used to predict the state variables $X_{k}$ until the next measurement $z_{k}$ is available.
For the Kalman filtering to work, the state covariance $P_{k}$ must also be estimated somehow from the last known state covariance$\;{\hat{P}}_{k}$. In the UKF the unscented transformation is used to estimate this state covariance.
The idea is to use a collection (2n+1, where n is the number of state variable) of well‐chosen system states [22], based on the last known state and state covariance and then propagate/predict the system state from each of those so called sigma points. The predicted system state $X_{k}$ is then the weighted average of these 2n+1 predictions. The estimated covariance $P_{k}$ is essentially the weighted covariance of the same predictions.

- **Correction step (measurement update)**:

Whenever a measurement is available, the predicted state values $X_{k}$ are combined with the measured values $z_{k}$ to provide corrected or filtered state estimates$\;{\hat{X}}_{k}$. For this reason the Kalman gain matrix $K_{k}$ must be calculated:
  (10) $$K_{k}=\;\frac{P_{k}\cdot H^{T}}{H\cdot P_{k}\cdot H^{T}+R}$$

Here *R* is the measurement error, *H* is the Jacobian matrix of the measurement model h ( ) and *P_k_* is the already mentioned estimated state covariance matrix of the prediction. The corrected or filtered system state ${\hat{X}}_{k}$ is essentially the weighted average of the predicted system state and the measurement with the Kalman gain as weight:

(11) $${\hat{X}}_{k}=X_{k}\;+K_{k}\cdot \left(z_{k}-h\left(X_{k}\right)\right)$$

The filtered state covariance ${\hat{P}}_{k}$ is updated based on the estimated state covariance $P_{k}$, the Kalman gain $K_{k}$ and the process noise covariance matrixQ:

(12) $${\hat{P}}_{k}=\;P_{k}-\left(K_{k}\cdot H\cdot P_{k}\right)+Q\cdot \Delta t-\left(K_{k}\cdot H\cdot Q\cdot \Delta t\right)$$

Δt is the time difference to the last known state/measurement.

The filtered values ${\hat{X}}_{k}$ and ${\hat{P}}_{k}$ can then be used as initial conditions for the next prediction/ simulation of the process as well as for the estimation of the covariance until the next measurement is obtained and everything repeats again.

The reliability and quality of a Kalman filter estimator can be evaluated by observabilty analyses, the theory of which has been established previously [21, 22]. Observabilty analysis provides an assessment of the theoretical possibility of estimating the state variables or parameters of the system from the available measurements (sensors) [23]. Due to Salau et al. [24], if the idea is to use the smallest number of sensors in order to simultaneously estimate the state and parameters of a system, using the Kalman filter makes it impossible to find complete system observabilty. For instance, in the process considered here, the two additional differential equations for parameter estimation of *μ*_max,G_ and *μ*_max,E_ produces a Jacobian matrix with corresponding row elements equal to null and therefore the observability is not given. However, due to the chosen process model with low correlation of the state variables, the observabilty of the process is guaranteed, since in this case, diagonal time‐invariant matrices Q and R can be successfully applied, which makes the UKF tuning considerably simple. A more comprehensive description about implementation of the UKF algorithm as well as the observabilty analysis can be found in [25, 26, 27, 28].

In this study, a continuous‐discrete UKF is used, i.e., a continuous time update and a discrete‐time measurement update. Table 1 presents the initial values for the UKF filter as well as the parameters of the model.

**TABLE 1:** Initial conditions for the mathematical model as well as the unscented Kalman filter

Parameter	Description	Value					
X_t = 0_	Initial biomass concentration	BC1	2.48 g/L	BC2	2.44 g/L	BC3	2.6 g/L
G_t = 0_	Initial glucose concentration	BC1	9 g/L	BC2	5 g/L	BC3	2.85 g/L
E_t = 0_	Initial ethanol concentration	BC1	0.1 g/L	BC2	0.18 g/L	BC3	0.25 g/L
$\mu_{\max,G}$	Initial maximal growth rate on glucose	BC1	0.16 h^−1^	BC2	0.18 h^−1^	BC3	0.14 g/h
$\mu_{\max,E}$	Initial maximal growth rate on ethanol	BC1	0.007 h^−1^	BC2	0.004 h^−1^	BC3	0.008 h^−1^
Y_X/G_	conversion from glucose to biomass	0.175 g_x_ g_G_^−1^					
Y_E/G_	conversion from ethanol to glucose	0.473 g_E_ g_G_^−1^					
Y_X/E_	conversion from biomass to ethanol	0.598 g_X_ g_E_^−1^					
K_G_	Monod constant for glucose	0.01 g/L					
K_E_	Monod constant for ethanol	0.01 g/L					
α	Unscented transformation constant	1e^−3^					
n	Unscented transformation constant	0					
β	Unscented transformation constant	2					
R	Measurement noise variance	0.0225 g^2^L^−2^					
Q	Process noise covariance matrix	diag (0.001 $g^{2}L^{2}h^{-1}$ 0.001 $g^{2}L^{2}h^{-1}$ 0.001 $g^{2}L^{2}h^{-1}$ 0.005 h^−3^ 0.005 h^−3^)					
P_0	Initial process estimation covariance matrix	diag (0 0 0 0 0)					
H	Observation matrix	(0 0 1 0 0)					

The UKF was implemented using the software Matlab R2019a (version 9.6.0); for all calculations a normal office PC (Intel CoreR i5 8500 with 8 GiB of RAM) with Window 10 was used. For the simulation, the system of in total 5 differential equations was solved numerically using the explicit, Runge‐Kutta based ode45 method from Matlab.

### 2.5 Off‐line measurements

Samples for analysing the concentrations of biomass, glucose, and ethanol were regularly taken from the bioreactor and put into pre‐weighed and pre‐dried micro‐centrifuge tubes. Cell dry weight was determined by centrifugation (Universal 16 R, Hettich Zentrifugen GmbH & Co. KG, Tuttlingen, Germany) of a sample with 1.5 mL (2 times) at 14,000 rpm for 10 min at 4°C. The wet cells were let in a drying oven at 103°C for 24 h. Subsequently, they were cooled down for 30 min before weighing. The supernatant of the samples after the centrifugation was examined by HPLC (ProStar, Variant, Walnut Creek, CA, USA) to determine the glucose and ethanol concentrations. Firstly, the supernatant was filtrated with pore size filter, 0.45 µm, polypropylene membrane (VWR, Darmstadt, Germany), then 20 µL was injected into a Rezex ROA‐organic acid H+ (8%) column (Phenomenex, Aschaffenburg, Germany) and operated at 70°C with 5 mM H_2_SO_4_ as an eluent at 0.6 mL/min flow rate. The concentrations of glucose and ethanol were calculated by Galaxie software (Varian, Walnut Creek, CA, USA).

In order to evaluate the performance of the UKF algorithm with respect to the accuracy of predicting ethanol concentrations, the root‐mean square error (RMSE) between the predicted ethanol concentration with the UKF and the off‐line ethanol concentrations was calculated and compared to the RMSE between the on‐line measured ethanol concentration and off‐line ethanol concentrations. Furthermore, the percentages standard error (SE) with respect to the maximum ethanol concentration, for the predicted ethanol concentrations from the UKF as well as the on‐line ethanol concentrations were calculated. RMSE and SE are calculated according to the following equations:

(8) $$MSE=\sqrt{\sum_{i\;=\;1}^{N}\;\frac{{\left({\hat{Y}}_{i}-\;Y_{i}\right)}^{2}}{N}}$$

(9) $$SE\;\;\left(\%\right)=\;\frac{\sqrt{\sum_{i\;=\;1}^{N}\;\frac{{\left({\hat{Y}}_{i}-\;Y_{i}\right)}^{2}}{N}}}{Y_{max}}\cdot 100\%$$

${\hat{Y}}_{i}$ represents the predicted ethanol concentration from the UKF algorithm or the measured on‐line ethanol concentration, $Y_{i}$ is the concentration determined by the off‐line values, N stands for the measurement count and $Y_{max}$ is the maximum ethanol concentration in the corresponding off‐line data.

To evaluate the accuracy of the estimated biomass and glucose concentrations from the UKF algorithm, the RMSE between the predicted values and the off‐line values as well as the SE with respect to the maximum off‐line value were calculated.

## 3 RESULTS AND DISCUSSION

**FIGURE 2:**
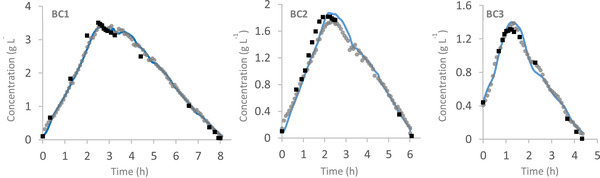
Measured ethanol concentration with the gas sensor array (grey dots), estimated ethanol concentration with UKF (blue curve) and off‐line ethanol concentration (black squares) during all three cultivations (BC1 – BC3)

The UKF presented in section 2 is used for continuous estimation of ethanol concentration on the basis of infrequent on‐line ethanol measurements from the gas sensor array. The UKF algorithm was also used for estimation of biomass and glucose as well as estimating the maximum growth rate on glucose and ethanol. The algorithm was validated on three cultivations with different initial conditions. Figure 2 presents the performance of the UKF for the estimation of ethanol concentration during three cultivation runs.

Figure 2 shows the estimates of the concentrations of ethanol in the bioreactor computed using the UKF (blue curve) together with the on‐line measured ethanol concentrations (grey dots) and the HPLC off‐line ethanol concentrations (black squares). Note that the HPLC off‐line values were not used during the estimation of the state variables, and are only taken to show that the estimates are accurate.

When a deviation of the on‐line measured and estimated values are present, the estimated values are shifting to the measured ones, indicating that the measured data are not dominating the estimation. For instance, in BC2 between 2 and 3 h cultivation time, there is a significant difference between the measured values and the off‐line values which can be caused by several factors including temperature fluctuations in the ethanol measurement chamber, an inappropriate determination of the base line, or electrical noise in the sensor circuit. Due to the fact that the process model fits to the off‐line values, it could be stated that the on‐line measured values are not accurate. In this case the UKF predicted values are a compromise of the process model and the on‐line measured values which leads to much more accurate predictions. The accuracy of the UKF regarding ethanol prediction was evaluated by comparing the RMSEP and SEP of the estimated (filtered) ethanol concentration with the non‐filter ones (the measured ethanol concentration), and the results are presented in Table 2.

**TABLE 2:** Comparison of off‐line measured values and UKF estimated ethanol concentration

	Off‐line measured values		UKF estimated values	
Cultivation	RMSEP	SEP	RMSEP	SEP
BC1	0.63 g/L	5.5%	0.15 g/L	4%
BC2	0.16 g/L	9.5%	0.08 g/L	4.5%
BC3	0.08 g/L	6.5%	0.09 g/L	4.5%

Table 1 shows the performance of the UKF compared with the case where the ethanol concentrations were measured off‐line. In BC2 the RMSEP and SEP of the off‐line measured ethanol concentration is considerably larger compared to the filtered ones. In BC1 and BC3 the UKF slightly increased the accuracy of the ethanol concentrations. However, the standard error of estimated ethanol concentration with the UKF during all cultivations is below 5% which is a decent value.

**FIGURE 3:**
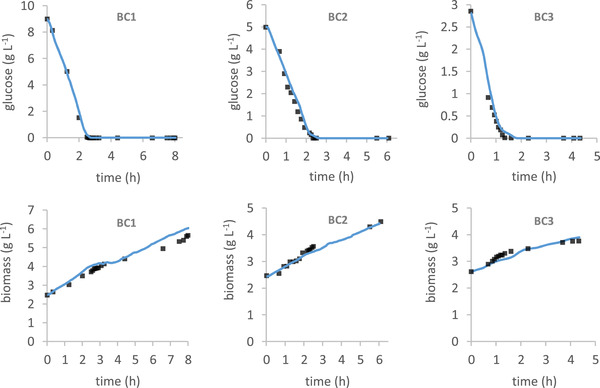
Estimated biomass and glucose concentrations with UKF (solid line) and off‐line biomass and glucose concentrations (black squares) during all three cultivations (BC1 – BC3)

The UKF is also able to accurately estimate the concentrations of biomass and glucose during the cultivations. Figure 3 presents the estimated values as well as the off‐line values of biomass and glucose for BC1 – BC3. In BC1 the estimates of the biomass concentration between 6 and 8 h cultivation time, deviate from the off‐line values, however their evaluation is almost the same. This might be due to faulty sample handling or measurement errors.

The data in Figure 3 indicates that the typical diauxic growth pattern of baker's yeast on glucose is observed. First the glucose is consumed and biomass as well as ethanol are produced, then ethanol is converted to biomass. The off‐line measurements and its corresponding estimated values fit quite well together as can be seen in Table 3.

**TABLE 3:** RMSEP and SEP for glucose and biomass

	Biomass		Glucose	
Cultivation	RMSEP	SEP	RMSEP	SEP
BC1	0.29 g/L	9%	0.13 g/L	1.7%
BC2	0.09 g/L	5%	0.16 g/L	4%
BC3	0.1 g/L	5%	0.16 g/L	4%

As illustrated above, the UKF is able to accurately predict the state variables in all three cultivations, based on the available infrequent on‐line ethanol measurements. However, as indicated in Figure 2 and Table 2, the measured ethanol concentration is not noisy, therefore an accurate estimation of the state variables by the UKF is not far from expectation. Therefore, in order to check the prediction ability of the UKF algorithm when the measurement data are noisy, the measured ethanol values were artificially distorted by random noise and the UKF algorithm was performed.

**FIGURE 4:**
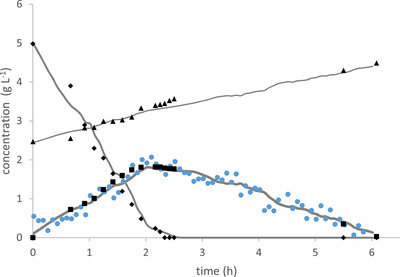
Artificially distorted measured ethanol concentration with random noise (●), estimated ethanol concentration with UKF (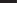), off‐line ethanol concentration (black squares), estimated glucose concentration with UKF (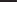), off‐line glucose concentration (black diamonds), estimated biomass concentration with UKF (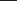), off‐line biomass concentration (black triangles) in BC2

Figure 4 shows the estimated as well as the off‐line values of state variables of BC2, by the UKF with considering noisy ethanol measurements. As it can be seen, even if the measurement values are artificially distorted by random noise, the UKF does not show much different results in predicting the state variables (SEP for all state variables is below 6%). The results for the other two cultivation data records are qualitatively the same, and are thus not repeated here.

As already stated previously, the UKF algorithm was also used for estimating the maximal specific growth rates. To prove the capability of the UKF for estimating the maximal specific growth rates, different starting values were chosen for these parameters in each cultivation. These values are chosen according to a rough estimate of these parameters.

**FIGURE 5:**
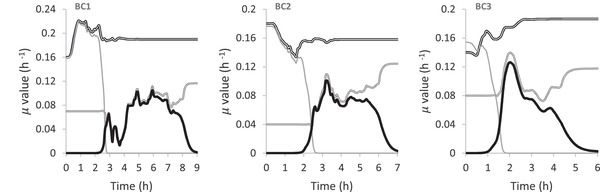
Estimated maximum specific growth rates with respect to glucose *μ*_max,G_ (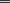) and ethanol *μ*_max,E_(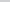) as well as the specific growth rates *μ*_G_ (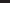) and *μ*_E_ (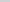) for glucose and ethanol respectively

Figure 5 presents the estimated maximum specific growth rates with respect to glucose *μ*_max,G_ and ethanol *μ*_max,E_ as well as specific growth rates itself for glucose *μ*_G_ and ethanol *μ*_E_ across all three cultivations.

In BC1 the *μ*_max,G_ and *μ*_G_ are increasing sharply short after the inoculation starts, this indicates that the chosen starting values are lower than the actual values, therefore the UKF algorithm converges to the true values. When the glucose is almost depleted, the transition from glucose to ethanol as substrate takes place, therefore *μ*_max,E_ and *μ*_E_ would start to increase. However, shortly before glucose is completely depleted, *μ*_max,G_ increases which results in the decrease of *μ*_E_, therefore the UKF increases the *μ*_max,E_ and *μ*_E_ to compensate the under estimation of *μ*_E_. According to the typical Monod behaviour, before ethanol is depleted, due to the low substrate concentration, *μ*_max,E_ should be almost constant while *μ*_E_ should be increasing. However this is not observed in BC1 which is due to the fluctuation of the measured and estimated ethanol concentration which can be seen between 4 and 7 h of cultivation time.

In BC2, after inoculation, the specific growth rates and its maximum values with respect to glucose are increasing slightly. This indicates that the chosen starting values are not far from the actual values. Accordingly, in BC3, the specific growth rates and its maximum values with respect to glucose are decreasing after the inoculation and shortly thereafter they increase again. This indicates that the chosen starting values are lower than the actual values, nevertheless the UKF algorithm converges them to reasonable values.

The high sensitivity of the estimated values due to the measurement noise variance and the process noise variance can be observed by comparing the estimated growth rates from BC1 to BC3. In BC2 and BC3, the measured and estimated ethanol concentrations shows less fluctuation compared to the ones from BC2, therefore the estimated values for the specific growth rates show less fluctuating compared to the ones from BC1.

**FIGURE 6:**
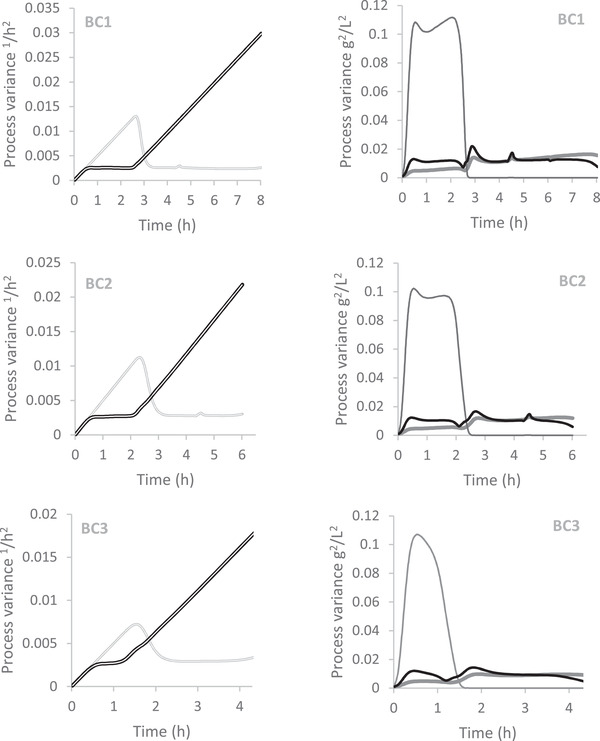
Estimated error variance of the maximal specific growth rates (*μ*_max,G_ (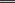) and *μ*_max,E_ (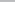)) and estimated error variance of the process variables (biomass (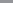), glucose (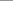) and ethanol (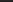))

In Figure 6 the estimation error variances of the process variables and the maximal specific growth rate with respect to glucose and ethanol are presented. The initial values of the variances are all set to zero. All values seem to be very much reproducible with respect to all three cultivation runs. During the glucose phase the estimation error variance values of glucose are much higher compared to the ones of biomass and ethanol. The values are roughly one order of magnitude higher. The reason is, that the ration of the change of ethanol and glucose with time during the glucose phase is the ration of the yield coefficients Y_GX_/Y_GE_ whose value is 0.37 g g^−1^. Therefore, the change in glucose is much higher (2.7 times) which causes a larger error and a larger variance. If one considers the variance of glucose as 0.1 g_2_ L^−2^ and ethanol as 0.01 g_2_ L^−2^ then the square root is 0.316 g/L and 0.1 g/L respectively. If one calculate the ration of error of ethanol by the error of glucose 0.1/0.316 then almost the same values is obtained as the ration of the yield coefficients. During the ethanol phase the estimation error variance of glucose become small, because no increase in ethanol can be detected and therefore no glucose is present. The variance of biomass and ethanol are stable throughout the cultivation. The variances of the of maximal growth rate on glucose are increasing fast to a constant value during the glucose phase and are increasing constantly during ethanol phase. The corresponding values with respect to ethanol behave in the same manner but inverted. If no measurement information of growth on the substrate is present, the estimation error variance is just increasing constantly, however the variance decreased clearly when growth occurred on that substrate. The constant values as well as the slope during increasing are the same.

## 4 CONCLUDING REMARKS

The design of monitoring and control algorithms to improve the performance of bioprocesses is of major importance. However, it is often difficult to find inexpensive and robust commercially available sensors that allow real‐time monitoring of important process variables, such as the biomass and substrate concentrations as well as the growth rates. Therefore, the development of software sensors is of paramount importance.

In this work, a dynamic nonlinear model was used and an unscented Kalman filter algorithm was implemented for parameter and state estimation during *S. cerevisiae* batch cultivaton. The proposed UKF algorithm only requires on‐line data from infrequent ethanol measurements together with the yield coefficients of the process model.

Three batch cultivations with different initial conditions were conducted in order to analyse the behaviour and performance of the UKF. The result obtained showed that with the proposed UKF algorithms, it was possible to estimate the specific growth rates as well as continuous ethanol, glucose and biomass concentrations with great accuracy, during the cultivation process. In order to check the quality margin for estimation with respect to presented noise in the measured on‐line ethanol values, a simulation was preformed; as a conclusion, the UKF algorithm is still able to predict the parameters and state variables, if the noise is about less than 10%.

The proposed UKF algorithm can be used for comprehensive monitoring of the baker's yeast batch fermentation process as well as for design and implementation of control strategies for the fed‐batch fermentation process of the baker's yeast.

## CONFLICT OF INTEREST

The authors have declared no conflict of interest.

## Data Availability

The data used to support the findings of this study are available from the corresponding author upon reasonable request.
